# Neonatal oxytocin gives the tempo of social and feeding behaviors

**DOI:** 10.3389/fnmol.2022.1071719

**Published:** 2022-12-13

**Authors:** Françoise Muscatelli, Valery Matarazzo, Bice Chini

**Affiliations:** ^1^Institut de Neurobiologie de la Méditerranée (INMED), INSERM, Aix Marseille Université, Marseille, France; ^2^Institute of Neuroscience, National Research Council (CNR), Vedano al Lambro, Italy and NeuroMI Milan Center for Neuroscience, University of Milano-Bicocca, Milan, Italy

**Keywords:** oxytocin, neurodevelopment, suckling, social interaction, Prader-Willi syndrome, Schaaf-Yang syndrome, autism

## Abstract

The nonapeptide oxytocin (OT) is a master regulator of the social brain in early infancy, adolescence, and adult life. Here, we review the postnatal dynamic development of OT-system as well as early-life OT functions that are essential for shaping social behaviors. We specifically address the role of OT in neonates, focusing on its role in modulating/adapting sensory input and feeding behavior; both processes are involved in the establishing mother-infant bond, a crucial event for structuring all future social interactions. In patients and rodent models of Prader-Willi and Schaaf-Yang syndromes, two neurodevelopmental diseases characterized by autism-related features, sensory impairments, and feeding difficulties in early infancy are linked to an alteration of OT-system. Successful preclinical studies in mice and a phase I/II clinical trial in Prader-Willi babies constitute a proof of concept that OT-treatment in early life not only improves suckling deficit but has also a positive long-term effect on learning and social behavior. We propose that in early postnatal life, OT plays a pivotal role in stimulating and coordinating the maturation of neuronal networks controlling feeding behavior and the first social interactions. Consequently, OT therapy might be considered to improve feeding behavior and, all over the life, social cognition, and learning capabilities.

## Highlights

– Actors of oxytocin system are dynamically regulated during early postnatal development.
– Oxytocin is necessary to integrate early sensory experiences and shape neuronal circuits.
– In mothers and neonates, oxytocin is critical for suckling and to establish the infant-mother bond and first social interactions.
– Early oxytocin treatment improves suckling and has positive long-term effects on social interactions.
– Suckling alterations might be a physiological marker for early diagnostic of ASD and indication of OT-treatment.

## Introduction

The perinatal period is critical for health and behavior later on life. The idea that the cause of a disease could be related to this critical period has been developed 30 years ago in the concept called “Primal Health research” (for review, Odent, 2001) that is now abandoned despite its relevance. The perinatal period is critical for pathologies related to feeding disorders or to sociability. An important factor to consider during this “primal period” is the neuroendocrine system (Lesage et al., 1996). The central oxytocin (OT) system, which projects to limbic and other central brain areas, appears to be a key regulator of the evolution and expression of different types of social systems in many species (for reviews: Choleris et al., 2009; Heinrichs et al., 2009; Insel, 2010). The current view is that OT modulates the coupling of central neuronal networks that process sensory cues activated during social interactions (Johnson and Young, 2017). More generally OT has been proposed as an allostatic hormone, acting in the brain and in peripheral organs, where it modulates both social and non-social vital behaviors by maintaining stability through changing environments (Quintana and Guastella, 2020). However, these assignments have been applied to the role of OT at adulthood.

During the last years, the scientific community studied the dynamic development of the neural OT circuitry. They have highlighted that how and when the neural circuit of the OT-system is organized in early development might have important consequences on behavior, a hypothesis indicates the “organizational effects of OT” (Eaton et al., 2012; Miller and Caldwell, 2015). More recently, studies revealed that an impact on OT-system ontogenesis during early-life period affects long-term behavioral expression (Onaka and Takayanagi, 2021). Furthermore, it is striking that the distribution of OT-receptor (OTR) in the brain undergoes dynamic changes in expression through the postnatal development with a peak of expression in the early infancy (in rodents and humans) and that this distribution correlates with brain regions controlling the sensory modalities primarily used to initiate social interactions (Hammock and Levitt, 2013; Tamborski et al., 2016).

Here, we discuss the early postnatal life role of OT shaping the sensory inputs and stimulating suckling, both contributing to the first social behaviors *via* the infant-mother bond. We also report the positive effects, in particular the long-term effects, of an early-life OT-treatment in neurodevelopmental diseases (NDD) such as Schaaf-Yang and Prader-Willi syndromes.

## Oxytocin neurons and oxytocin receptors are dynamically regulated through development

OT-system is defined by OT-producing cells and OT-target cells that together determine the qualitative and quantitative characteristics of OT responses. The action of OT is then controlled by the release of OT, the quantity and spatio-temporal expression of OT-binding sites on the target cells (OT-receptors and vasopressin receptors), and the intra-cellular signaling pathways triggered by OT. Many reviews have described the source of OT production (Althammer and Grinevich, 2017), the mechanisms of release (Johnson and Young, 2017; Brown et al., 2020), and the cellular signaling pathways *via* the OT-binding sites (Busnelli and Chini, 2018; Jurek and Neumann, 2018). Here, we will focus on developmental setup of the OT-system.

OT is produced by hypothalamic neurons of several regions: the Supraoptic Nucleus (SON), the Periventricular preoptic nucleus (PvPO) the Paraventricular Nucleus (PVN), the Accessory Nuclei (AN), the Bed Nucleus of the Stria Terminalis (BNST) and in a recently identified region named Antero-Lateral Preopticarea (ALPO; Soumier et al., 2022). Recently, a comprehensive map of OT/AVP neurons and projections has been generated in the mouse brain during development (Madrigal and Jurado, 2021) and at adulthood.^1^ OT and AVP expression show distinct developmental dynamics in the mouse brain, with developmental maturation of AVP preceding that of OT (Madrigal and Jurado, 2021). The number of OT cells selectively increases during a critical window of postnatal development in several hypothalamic regions: in the PvPO and PVN, as well as in ALPO (Soumier et al., 2022). Noticeably, AVP-expressing cells in the different hypothalamic nuclei (PVN, SON, Medial Preoptic Nucleus, Tuberal Nucleus), as well as in the extra-hypothalamic regions, such as the Medial Amygdala (MEA) and BNST, remain stable over time, from birth to adulthood (Soumier et al., 2022). Interestingly, in most of hypothalamic nuclei, there is a high percentage of neurons coexpressing OT and AVP that decreases at adulthood (Madrigal and Jurado, 2021).

OTR is a seven-transmembrane segment G protein-coupled receptor (GPCR). To date only one subtype of OTR has been described which is closely related to the three vasopressin (AVP) receptors, OT binds also AVPR-1a with a lower affinity. OTR mapping at the protein level is hampered by the lack of specific anti-OTR-antibody. Consequently, the distribution of OTR expression has been analyzed using different experimental approaches, such as receptor binding of radiolabeled OT analogs on tissue sections, *in situ* hybridization and transcriptomic analysis, and the use of transgenic mice expressing a fluorescent reporter under the control of the OTR promoter. In adult rodents, a comprehensive list of brain areas expressing OTR has been reported in the rat and mouse brain (Jurek and Neumann, 2018). The four main and best-studied OTR-expressing brain regions are the hypothalamus, prefrontal cortex, hippocampus, and amygdala (Jurek and Neumann, 2018; Carter et al., 2020). Son et al. (2022) have shown that there is no significant correlation comparing the quantity of OT projections and OTR expression across the whole brain, suggesting important indirect OT-signaling in OTR-expressing areas.

Comparative analysis of OTR distribution in brain rodents (i.e., prairie voles, rats, and mice) revealed species, sex, experiences, and developmental differences in OTR expression (Vaidyanathan and Hammock, 2017). In rats, using radiolabeled OT, it was shown that OTRs progressively appear in several brain regions throughout the development, being first detected in the presumptive embryonic region of the vagal motor nucleus from the embryonic day 14.5 (E14.5), and reaching a well-defined “infant” pattern of distribution after the Postnatal day 10 (PND10). OTR expression then follows a differential time course depending on the brain structure considered. Interestingly, during the early postnatal period some transient expressions are detected in several brain areas (Tribollet et al., 1989, 1991; Yoshimura et al., 1996). After PND13, the density of OTR drops sharply in several brain regions, and expression in new brain areas appears; this is referred to as the first transition to the adult pattern, which is almost reached at PND18. Around weaning and beyond, a second transition takes place that is characterized by a new remodeling of OTR expression, disappearing from some areas and increasing in others. Finally, the adult pattern of OTR expression is established at PND60-90 (Vaidyanathan and Hammock, 2017; Muscatelli et al., 2018).

A similar radioligand binding approach was performed in the mouse brain and reported an early detection of OT-binding sites atE16 (Tamborski et al., 2016), with a peak around 2 weeks after birth followed by a decrease in OT-binding sites in all brain regions thereafter (Hammock and Levitt, 2013); such a strong a transient expression of OTR around PND14 is particularly detected in different cortical regions (Hammock and Levitt, 2013). Yongsoo Kim’s lab used a fluorescent reporter mouse model (i.e., OTR Venus mice) and established a publicly available brain-wide map of the OTR in mice during postnatal development^2^ (Newmaster et al., 2020). The drawback of this map is that it does not allow to visualize transitory expression. Studies in prairie voles also showed a dynamical expression profile of OTR over time (Prounis et al., 2018).

In conclusion, in all rodents examined (i.e., mice, rats, and prairie voles), *Otr* mRNA and OT-binding sites are present in embryos, even though the mature form of OT is not detected before birth, and the peak of OTR expression is observed around 2 weeks after birth. The distribution of OTRs during brain development is different from that of adult brains and can be classified into three expression profiles: 1) clusters of neurons with constant expression, where OTR expression begins to be detected during development and is maintained throughout life, 2) sites with transient expression where OTR is only observed during a developmental time window and its expression is no longer detectable after that, and 3) neurons where OTR expression begins to be detected during puberty and is maintained throughout life (Vaidyanathan and Hammock, 2017). OTR expression is sexually dimorphic, with *Otr* mRNA expression generally higher in female than in male brains (Tamborski et al., 2016).

In the human brain, similarly to rodents, OTR expression begins to accelerate just before birth, with a peak level expression occurring during infancy (Rokicki et al., 2022). There is, however, a sexual dimorphism of OTR expression. In girls, a peak of expression is observed around birth, with a decrease in expression during childhood, and a dip during adolescence. In boys, compared with girls, the peak of OTR expression is delayed in early childhood and the difference in expression between brain regions is more pronounced (Rokicki et al., 2022). This study used genome-wide exon-level transcriptome approach to define OXTR transcript density in 16 brain regions through different ages (pre-natal to 82 years old) and using males and females. The study is robust but there is a lack of quantification of the active transmembrane OT-receptors.

OTR are also strongly detected in the peripheral tissues of neonatal mice and prairie voles. In particular, OTR is transiently and highly expressed in the oro-facial region of mouse with marked sex and species differences (Greenwood and Hammock, 2017).

Noticeably, the distribution of OT and vasopressin (VP) receptors shows overlapping expression in many regions and specific non-overlapping expression in some brain structures as shown in the lemurs (Grebe et al., 2021). These two neural systems play a role in the same signaling pathway, and have either complementary or opposing functions depending on the species, sex, age, and social experiences.

The dynamic of OT release in postnatal development is poorly studied and, paradoxically, although there is a huge number of OTR in the infant brain, the synaptogenesis of OT neurons is not fully completed. Interestingly, Hoffiz et al. (2021) showed that birth triggers specifically c-Fos activation of VP and OT neurons from the suprachiasmatic (VP only), supraoptic, and paraventricular nuclei of the hypothalamus. These activations are high at 3 h postnatal and returned to baseline levels at P1.

**Figure 1:**
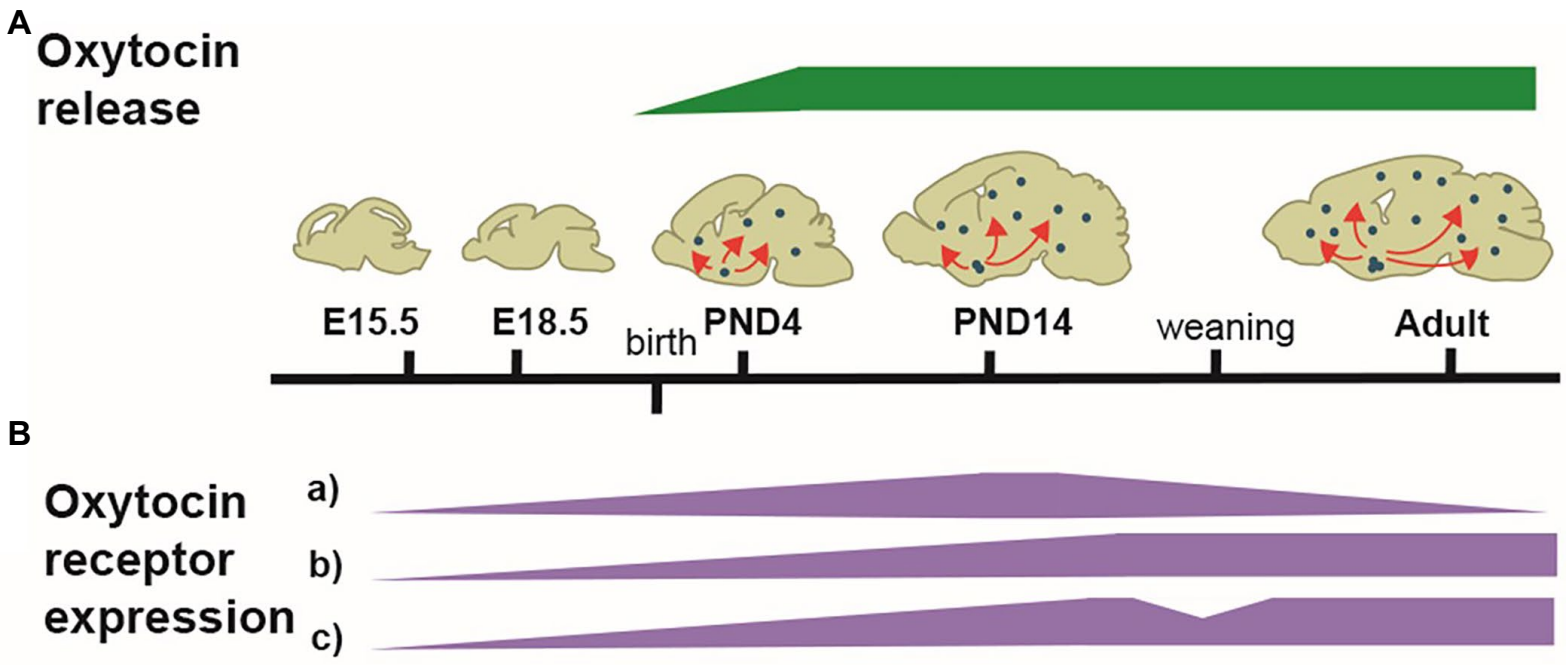
Illustration of the developmental dynamic of the OT-system. **(A)** Schematized patterns of OT produced in the hypothalamus and released in the forebrain (green). OT is first released at birth. **(B)** Schematized patterns of OTR expression (purple). From E14.5, OTRs progressively appear in several brain regions, with different dynamic patterns of expression. In many areas, OTRs pick between PND14-PND21, followed by either a decline (a) or a stabilization (b); in some areas, a reshaping of OTRs expression occurs at weaning, with a disappearance from some areas and an increase in others (c). The adult pattern of OTR expression is achieved at PND60-90.

In conclusion, the pattern of OT-system is highly dynamic throughout development, particularly from birth with transition phases at weaning time, puberty, and young adulthood (Figure 1).

## OT-system integrates sensory experiences in early life and shapes developmental neuronal circuits

At adulthood, the role of OT as a modulator of sensory processing in relation with the social behavior has been clearly shown for olfaction (Oettl et al., 2016; Oettl and Kelsch, 2018) and touch (Tang et al., 2020; Yu et al., 2022). OT has been shown to modulate pain perception (Poisbeau et al., 2018). In dams, OT modulates audition allowing them to recognize the vocalizations of their pups and to adapt maternal behavior accordingly (Schiavo et al., 2020).

During postnatal development, numerous studies show a role of OT-system as an integrator and modulator of sensory inputs, allowing to shape brain circuits and connectivity (Grinevich and Stoop, 2018). Interestingly, VP and OT neurons of the supraoptic and paraventricular nuclei of the hypothalamus were specifically activated in the 30 min following birth in mice and rats (Hoffiz et al., 2021).

Some of the pioneer studies showed that in rat (Nelson and Alberts, 1997; Lenz and Sengelaub, 2010), rabbit (Caba et al., 2003), or prairie voles pups (Barrett et al., 2015), mimicking parental licking stimulates the activity of hypothalamic OT neurons. In the rat, this stimulation also induced an increase in OT concentration in the spinal cord, suggesting that maternal licking may affect the maturation of sensory and autonomic centers (Lenz and Sengelaub, 2010). Affiliative stimuli, defined by gentle tactile stimuli of the pups during developmental period, activate also OT neurons (Walker et al., 2017). Maternal care (skin to skin contacts) stimulates the central OT in pups creating the conditions for inducing a preference for maternal odor (Kojima and Alberts, 2011a; Kojima et al., 2012) and for establishing a social affiliation in rat pup’s filial huddling preference (Kojima and Alberts, 2011a; Kojima et al., 2012). The increase in OT induced in pups by contact with the mother also facilitates the development of thermal-seeking huddling behavior (Kojima and Alberts, 2011b).

One of the most convincing demonstration of the sensory cues-dependent release of OT in the pups’ brain comes from experiments based on blocking early sensory stimulation (Zheng et al., 2014). Deprivation of sensory inputs, such as whisker deprivation, right after birth (ending at PND 14) results to reductions of the firing rates of OT PVN neurons and lower released of OT in the brain of juvenile mice (Zheng et al., 2014). This deprivation also affects other sensory cortices in the brain and caused a reduction in excitatory synaptic transmission across the other sensory cortices, demonstrating that OT promotes cross-modal, experience-dependent cortical development. Similar results were obtained in dark rearing. Injection of OT into the brains of sensory-deprived animals enabled them to recover this deficiency and improved the brain’s response to other sensory inputs. Conversely, increased sensory stimulation from birth through environmental enrichment increases at PND14 the level of OT, excitatory synaptic transmission in several sensory cortices, and corrects the effects of sensory deprivation. This study thus demonstrates that during the period of synaptogenesis, OT promotes excitatory synaptic transmission and, in sensory cortices, mediates early experience-dependent multimodal plasticity. Noticeably, this action of OT, which regulates excitatory synaptic transmission in pyramidal neurons of sensory cortices, is maximal around PND14 and ceases after PND18, revealing a sensitive/critical period (Zheng et al., 2014). Similarly, sensory experience regulates the expression of OTRs with a similar time course, an elevation at PND14, and essentially no change at PND18 (Maldonado et al., 2021). More generally, OTR expression in the pyramidal glutamatergic neurons of sensory cortex peaks around PND14 and drops significantly at PND21 (Hammock and Levitt, 2013; Newmaster et al., 2020; Maldonado et al., 2021). Then, at PND28, *OTR* expression is more important in GABAergic neurons, especially somatostatin interneurons (Zheng et al., 2014). Thus, a reduction in OTRs expression may promote the closure of the sensitive/critical period (Zheng et al., 2014).

In a similar manner, it has been shown that OT affects spontaneous activity patterns in the mouse developing visual cortex, recruiting Somatostatin interneurons and allowing to refine the synaptic connections in the visual cortex prior to eye opening (Maldonado et al., 2021). OT is also required in early infancy to impose the positive quality of olfactory imprinted memory in the nursed neonates, in which imprinted memory is associated with a pleasant mental state. Importantly, there is a critical period (first week of life) during which OT, by imposing positive quality on imprinted odor memory, contributes to smooth social interactions in the adult life (Inoue et al., 2021). Noticeably, during early infancy, the transient strong oro-facial expression of OTR (see above) suggests also an effect of OT-system in the peripheral control of sensory inputs. In our team, we have shown a role of OT in thermo-sensory reactivity of neonates. Indeed, in a cool environment, neonate mice emit ultrasonic vocalizations that trigger a “pup retrieval” behavior from their mothers. Inactivation of OT neurons prevents this thermo-sensory reactivity of neonates (Da Prato et al., 2022).

**Figure 2:**
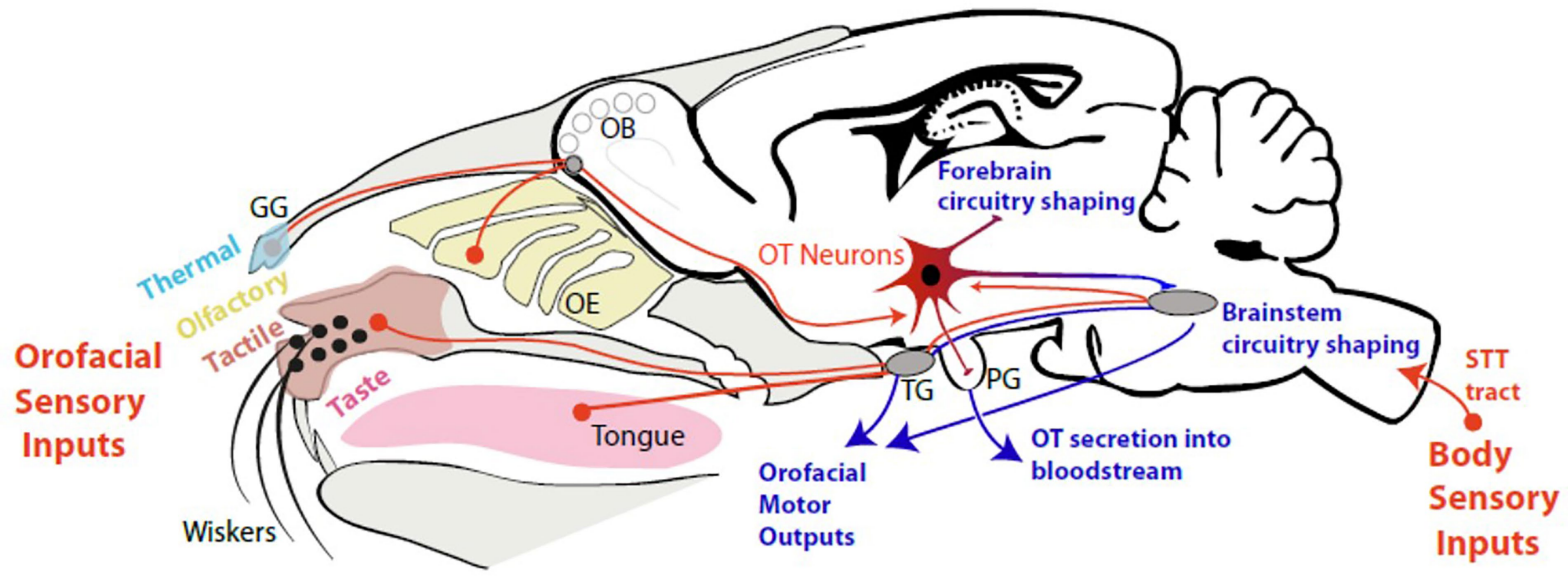
The oro-facial sensory inputs in neonates stimulate OT neurons. OB: Olfactory Bulb; OE: Olfactory Epithelium; OT: Oxytocin Neurons; GG: Grueneberg Ganglion; PG: Pituitary Gland; TG: Trigeminal Ganglion; STT: Spino-Thalamic tract.

Altogether, these data reveal a stimulation of OT-system in response to all types of sensory inputs (tactile, visual, olfactive, thermal), in particular those linked to the oro-facial stimulation, triggered at birth and during a critical period of time of infancy (Figure 2). In this period, sensory experience influences the production of OT and OT shapes neuronal circuits by modulating spontaneous and evoked activity.

## OT-system and sensory experiences in early life shape the first neonates-mother bond, the ground of social interactions

In all mammals, the mother-infant bond is the first social experience. Many studies report the role of OT in mother-infant bonding induced by lactation and maternal behavior in the mother (Nagasawa et al., 2012; Okabe et al., 2012). In addition, an important effect of maternally produced OT at the time of birth could protect the neonatal brain of rodents from birth stress by decreasing perinatal neuronal activity (Tyzio et al., 2014). On the other side, the role of OT produced by the newborn in stimulating bonding with its mother has been much less studied. The newborn’s OT-system is activated by parental care which is responsible for all of the sensory inputs described above (Paragraph 2); deficiencies in parental care, as it happens in maternal separation, impact the OT-system and alter social and emotional behaviors later on in adulthood (Onaka and Takayanagi, 2021).

### OT in the mother is required for lactation and shapes maternal behaviors

OT has gained considerable attention for its role in modulating bond formation on the mother side (Uvnas-Moberg and Eriksson, 1996). OT is a key component in the transition to motherhood, affecting molecular pathways that dampen stress reactivity, promote positive disposition, and regulate healthy maternal behaviors (Bell et al., 2014). Maternal breastfeeding behavior is vital for newborn’s access to food and survival, and to establish a solid bond between the mother and neonates. OT is well known for its crucial role in lactation and suckling (Uvnas-Moberg and Eriksson, 1996). Indeed, OT knockout mice, although able to deliver pups, have a complete failure of milk letdown (Nishimori et al., 1996) During lactation, milk synthesis is promoted by prolactin and oxytocin stimulates milk ejection. A study in lactating rats showed a switch of prolactin inhibition into excitation on OT neuron activity (Augustine et al., 2017). In addition, suckling activity by the pups stimulates central release of OT in the mother (Neumann et al., 1993), therefore providing a mean for stimulating milk production, and also reinforcing mother-infant bonding. Furthermore, mothers provide olfactory cues and signals that direct their unexperienced newborns to the nipple and optimize initial sucking success and, therefore, viability (Al Ain et al., 2013). In addition to control maternal behavior and milk letdown, dam’s OT contributes to the olfactory signals attracting pups toward the nipple. Indeed, the administration of OT in a mother whose nipples have been washed reinstates the attachment of the pups to the nipples; this suggests that a substance, which acts as an olfactory cue for the pups, may be excreted from the nipple area in response to circulating OT (Singh and Hofer, 1978). Newborn rats are strongly attracted to the odor of amniotic fluid when exposed to the nipple for the first feeding (Arias and Chotro, 2007). Similarly, in mouse, olfactory cues present in amniotic fluid and milk induce nipple grasping before the first suckling experience (Al Ain et al., 2013). During the following suckling, milk or maternal saliva can trigger sucking (Al Ain et al., 2013). In rabbits, a specific pheromone in milk is responsible for nipple grasping (Schaal et al., 2003).

The mother develops cross-modal sensory perception of olfactory, auditory, and tactile stimuli from the pups, which allows her to recognize her own pups and induces stereotyped maternal behavior (Nagasawa et al., 2012; Okabe et al., 2012). Although it has been known for decades that OT can trigger maternal behavior such as pup retrieval, nursing, or grooming a newborn (Pedersen and Prange Jr., 1979; Pedersen and Boccia, 2003; Rilling and Young, 2014), the neuronal encoding of this behavior has started to be deciphered only recently. First, given the important role of pup calls, Marlin et al. (2015) revealed that OT increases the sensitivity of auditory cortex neurons of the dam to mouse pup calls. A hypothalamo-cortical circuit involving a sub-population of OT neurons projecting from the PVN to large population of OTR-expressing interneurons located in the bilateral auditory cortex of female allows the dams or an OT-treated virgin female to disinhibit the auditory cortex firing after pup call stimulation and to facilitate pup retrieval (Marlin et al., 2015). Remarkably, it has also recently been shown that mouse alloparenting behavior might be acquired by social transmission and involves OT (Carcea et al., 2021). In the mother’s brain, OT is thought to amplify the neural circuits activated by signals from the pups, such as distress calls. This amplification would occur in brain regions important for learned aspects of maternal care, including the left auditory cortex. These precious alloparenting experiences acquired during co-housing (mother with virgin female) are likely to improve the quality of early maternal care when the virgins themselves have litters (Carcea et al., 2021).

### OT in neonates shapes the first social behaviors *via* the infant-mother bonds

Mother-infant bonding is not only a positive factor of newborn survival (Nowak et al., 2000), but also serves the newborn’s formation of functional neural circuits through sensory stimulation experiences (Sur and Rubenstein, 2005). From birth, mammalian neonates must efficiently interact with their mother to obtain care and food (Schaal et al., 2009). Olfactory, tactile, and thermal cues coming from the environment or the mother’s body promote sensory stimulations that elicit the neonates to undertake active, often stereotyped, behaviors such as nipple-searching, sucking, ultrasonic vocalization, wriggling calls for successful feeding and warming (Nowak et al., 2000; Grimsley et al., 2011). As described above, OT-system is active from birth and responds to social/sensory stimulation produced by the mother. The production and release of OT in the newborn is required for shaping these first sensory-motor functions and to establish a strong relationship with the mother.

### OT in neonates and the early feeding behavior/suckling

Mouse neonates suck milk after finding their mother’s nipples on their own. The olfactory function is essential for nipple-finding behavior and milk sucking. The stimulation of the tactile system (defined as the “trigeminal-whisker system”) cannot replace the absence of olfactory input in mouse neonates. (Hongo et al., 2000). However, it might participate since the olfactory and trigeminal systems interact; indeed, odorants stimulate the olfactory bulb but also the trigeminal nerve (Frasnelli et al., 2007). Alberts and Ronca (2012) proposed that, in rat neonates, mechanical and thermal sensory stimulations just before and during birth established the sufficient conditions for the maternal odor learning that guides newborn’s sucking responses. Thus, the hypothesis is that odors learned prenatally in association with perinatal stimuli around birth, become conditioned stimuli for nipple attachment (Pedersen et al., 1982; Alberts and Ronca, 2012). Thus, a sensitive period to learn odor-preference (of the mother) has been proposed, the neural circuit involved the olfactory bulb, the piriform cortex, the amygdala, and the locus coeruleus, that produces norepinephrine (NE) which is required to enhance odor-preference in this sensitive period (Sullivan, 2003). Today, studies have to be conducted to assess a role of OT in mother odor learning guiding newborn’s sucking responses. OT neurons are activated just after birth (Hoffiz et al., 2021) and there is a transient but strong expression of OTR around birth in several feeding-relevant peripheral regions of the face (Greenwood and Hammock, 2017), suggesting a role of OT in the integration of sensory inputs associated with suckling. Interestingly, rabbit pups are nursed and fed once every 24 h (unusual among mammals) and the OT neurons of the SON and PVN are differentially activated by sucking of milk and anogenital stroking, a sensory input from the mother to enhance suckling in pups (Caba et al., 2003). It should be noted that OT is present in milk and colostrum and thus regulates the intestinal development of newborns and protects their gut from inflammation (Klein et al., 2017).

Several publications suggest that OT is involved in motor outputs involved in sucking. Hypoglossal (XII) motoneurons innervate extrinsic and intrinsic muscles of the tongue and are necessary for sucking. Using brainstem slices of 5–9-day-old rats and whole-cell patch clamp recordings from XII motoneurons, Wrobel et al. (2010) proposed that VP and OT function as neuromodulators of the hypoglossal (XII) nucleus and may be part of neuronal networks underlying rhythmic tongue movements. These effects were mediated by vasopressin 1A (V1A) and OT-receptors present in XII motoneurons as well as in interneurons and premotor neurons, making synaptic contact with them.

**Figure 3:**
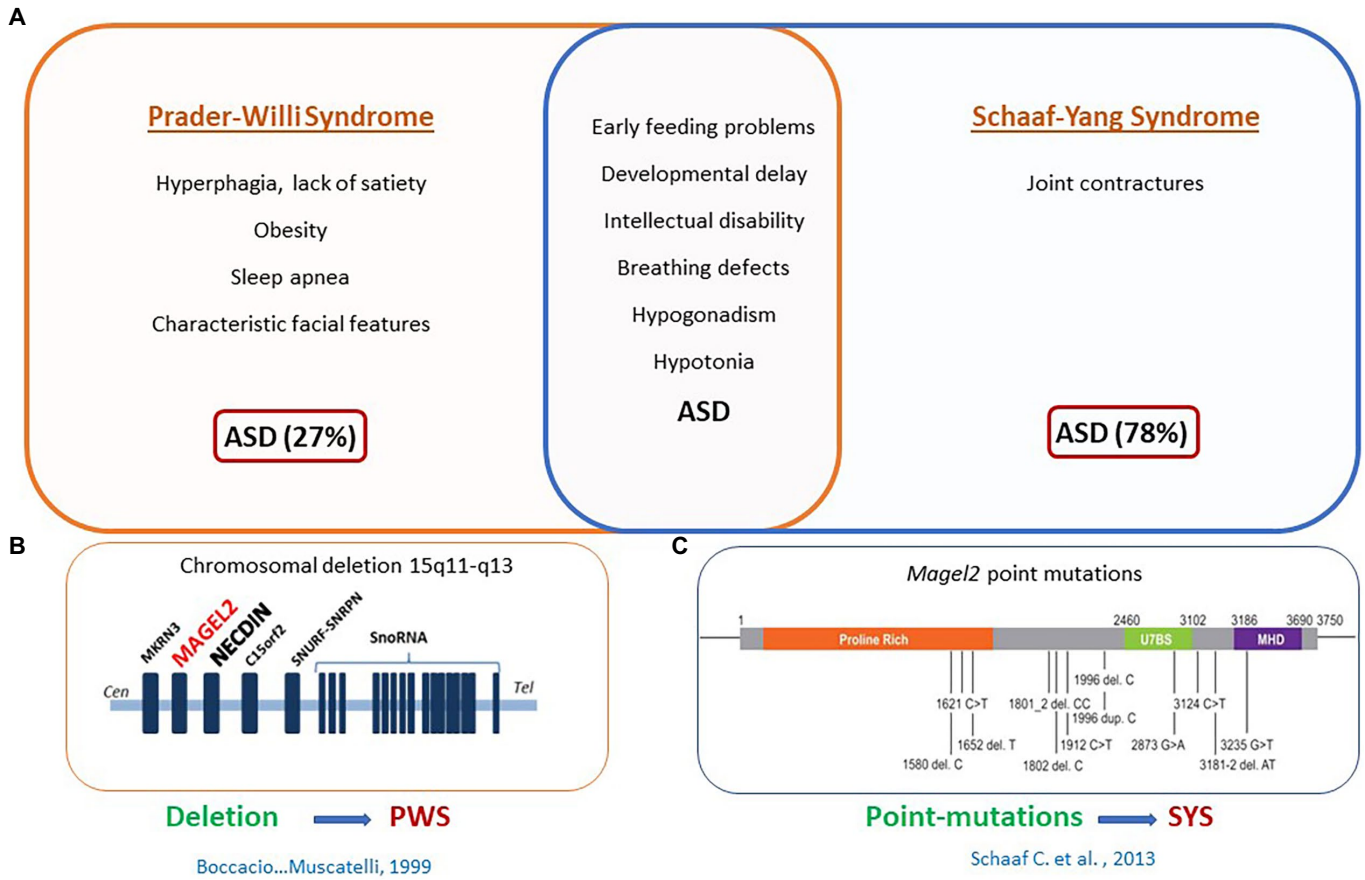
*MAGEL2* is involved in Prader-Willi syndrome (PWS) and Schaaf-yang syndrome (SYS). **(A)** Clinical symptoms specific to PWS or SYS or common to both syndromes. **(B)** Common chromosomal deletion observed in PWS. **(C)** Point mutations found in MAGEL2 gene of patients with SYS.

**Figure 4:**
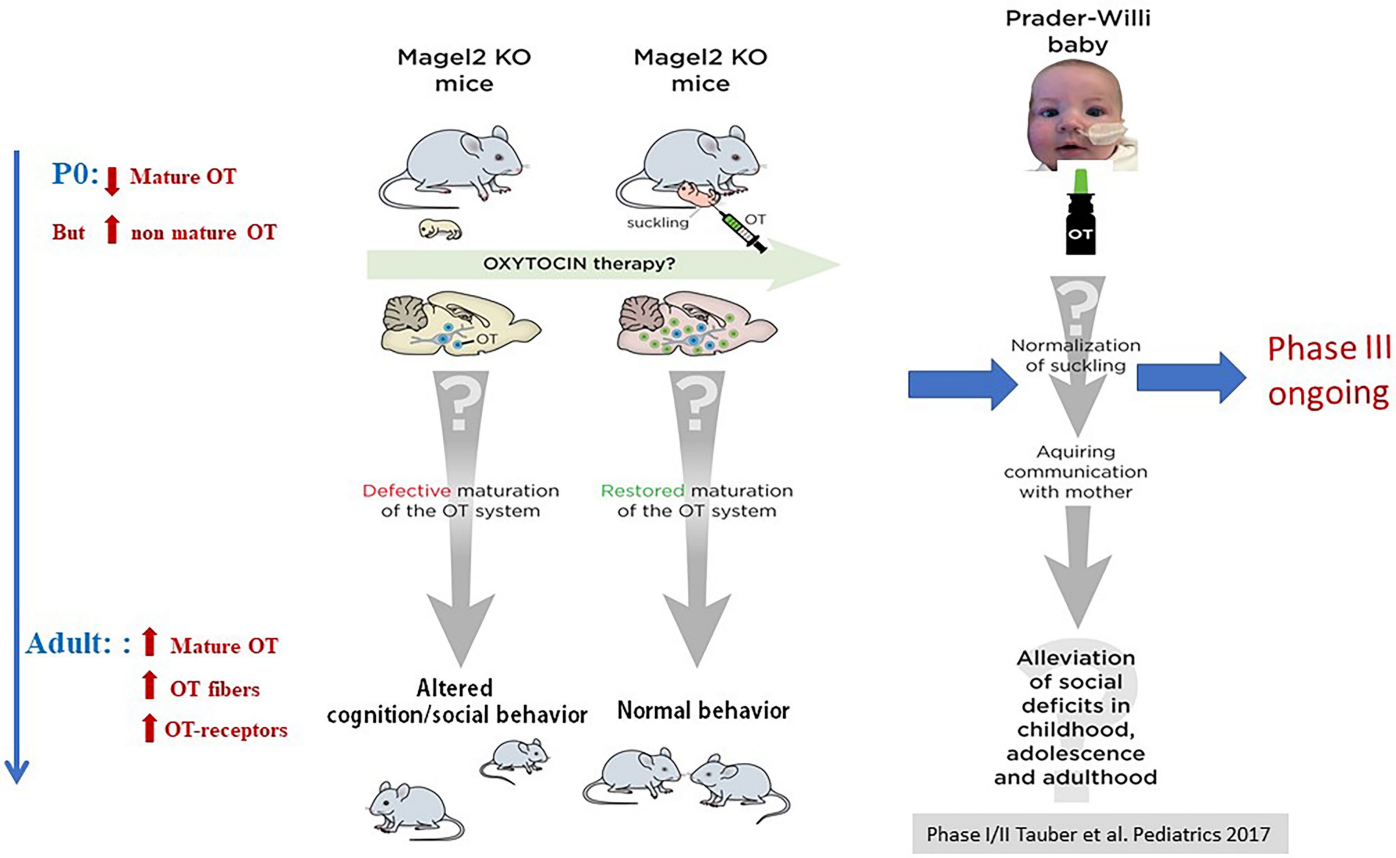
Alterations of OT-system and OT-rescue in *Magel2* KO mice and effects of OT intranasal administration in babies with PWS. Adapted from Muscatelli et al. (2018).

Perhaps the strongest demonstration for a role of OT in the newborn’s feeding behavior comes from studies in our laboratory initially devoted to characterize the phenotype of *Magel2* deficient mice. Deficiencies in the OT-system are implicated in neuropsychiatric diseases presenting an autism syndrome disorder, including Prader-Willi (PW) and Schaaf-Yang (SY) syndromes, characterized by impaired suckling at birth, requiring intubation feeding (Fountain and Schaaf, 2016; Figure 3). Both syndromes are associated with mutations in *MAGEL2* gene: either point mutations in SYS or a large chromosomal region including *MAGEL2* in PWS (Figure 3). Our team studied the *Magel2* KO mice, we observed the death of approximately 50% of the mutant mice within the first postnatal day due to deficiency in suckling activity and associated with low OT levels in the neonate hypothalamus (Schaller et al., 2010). This suggested that OT deficiency participate in the altered feeding behavior of *Magel2* knockouts, which was confirmed in further experiments. Indeed, injection of a specific potent OTR antagonist to wild-type pups 1–1.5 h after birth prevented suckling in approximately 50% of mouse neonates, which were found dead the day of the injection (Schaller et al., 2010). Interestingly, the same injection performed 12–24 h after birth had no effect on the pups. In addition, the feeding deficiency of *Magel2* knockouts was rescued by a single subcutaneous OT injection 3–5 h after birth (Schaller et al., 2010). In a phase 2 clinical study following on from the preclinical work, we have shown that intranasal administration of OT (during 1 week) in 18 Prader-Willi infants (4 weeks to 5 months old) improved and normalized sucking in 88% of babies. Sucking was assessed by the Neonatal Oro-Motor Scale and videofluoroscopy showing a great improvement of the motor outputs (Tauber et al., 2017). Therefore, these results indicate that manipulating the OT-system very early after birth could greatly impact on the initiation of suckling activity (including sucking) in *Magel2* KO newborn mice and Prader-Willi babies (Figure 4).

It is noteworthy that the role of OT in feeding behavior changes during development, as it stimulates milk production in the mother, stimulates feeding behavior in the neonate, and inhibits food intake in the adult. The underlying mechanisms that might support this developmental shift in role are currently unknown, but it is clear that the respective roles of maternally secreted OT and OT produced by infant brain will allow for a gradual tiling during the lactation period.

## Long-term effects of oxytocin produced in early life

### OT-system is disturbed in rodent and human NDD associated with autism spectrum disorders

OT-system is disrupted in several animal models of neurodevelopmental disorders characterized with autism-like phenotypes (Muscatelli et al., 2018; Wagner and Harony-Nicolas, 2018; Althammer et al., 2022). Indeed, mouse models, which are deficient for genes such as *Or (*Winslow et al., 2000; Ferguson et al., 2001), *Otr* (Takayanagi et al., 2005; Sala et al., 2011, 2013), or *ADP-ribosyl cyclase* (CD38) (Jin et al., 2007; Liu et al., 2008), show changes in social behavior that are indicative of autism spectrum disorders (ASD). On the other hand, several rodent models known to be models of ASD, indirectly show a deficit of the OT brain system (Wagner and Harony-Nicolas, 2018). These models result from the inactivation of genes such as *Fmr1, Cntnap2, Magel2, Oprm1, Shank3* (Bosch and Neumann, 2008), *Nlgn-3* (Lefevre and Sirigu, 2016), or from environmental exposure to valproic acid. Although most studies of OT-dependent social behaviors have been conducted in adulthood, there is compelling evidence for a key role of OT, in infancy, in shaping various social behaviors and traits (Bosch and Neumann, 2008; Eaton et al., 2012; Miller and Caldwell, 2015; Lefevre and Sirigu, 2016; Muscatelli et al., 2017). Lukas et al. (2010) have shown that if pups are exposed to early-life stress, such as maternal separation, the normal development of V1A-R and OTR binding in specific forebrain regions is altered. Such alterations could contribute to aggression (Lopatina et al., 2018; Reichova et al., 2021) and other altered social behaviors, such as sexual behavior or social cognition in adulthood. In humans, recent studies suggest a link between child abuse (Smearman et al., 2016) and ratings of parental care (Unternaehrer et al., 2015) with the methylation status of the OTR promoter, conditioning the levels of OTR expression.

An unbalanced excitation/inhibition ratio and an altered synaptic plasticity have been associated with most of these pathologies and OT, *via* the OTRs, controls GABA-mediated Excitatory/Inhibitory (E/I) ratio (Lopatina et al., 2018) and synaptic plasticity *via* synaptic molecules (Reichova et al., 2021). Along this line, evidence for a unifying role of OT in pathogenic mechanisms responsible for social impairments across a broad range of autism etiologies has been provided (Hornberg et al., 2020; Lewis et al., 2020).

In addition, early-life adverse experience impairs social behaviors and has long-term, sex-dependent effects on the OT-system, in particular the expression of OTR (Bales and Perkeybile, 2012; Veenema, 2012; Perkeybile et al., 2019; Lapp et al., 2020). It has also been demonstrated that, in mouse pups, sensory experience influences OT production and that OT shapes neuronal circuits by modulating spontaneous and evoked activity. For instance, transection of the infraorbital nerve at P3, a well-known model of whisker deprivation leading to loss of barrel structures and callosal connection in the somatosensory cortex, compromises social memory and spatial memory in adult mice (Zhang et al., 2016). In this context, the adult social memory deficit is associated with a reduced quantity of OT in the hypothalamus and could be partially restored by intranasal administration of OT (Zhang et al., 2016).

Together, these studies support a role for the OT-system in the early postnatal development of various brain regions, especially in cortex and hippocampus. The OT-system resulted to be at the same time a target and a mediator of early sensory functions; it is stimulated by sensory inputs and it mediates adaptative sensory and motor responses, *via* neuromodulation. The release of OT in infancy controls the quantity of OTR in the developing and adult brain and the E/I ratio *via*, partly, the GABAergic activity and the synaptic plasticity.

### Long-term effects of oxytocin treatment in infancy

A highly informative approach to investigate the early effects of the OT-system is to administrate OT in neonates after birth. In wild-type prairie voles, such administration has lasting effects on both, the OT-system and the social behavior (Bales and Carter, 2003a,b; Cushing and Kramer, 2005; Bales et al., 2007). Similarly, the effects of maternal OT administration on prairie vole offspring development have also been characterized (Bales and Carter, 2003b; Kenkel et al., 2019). All pups showed at adulthood an increased alloparental caregiving toward pups and an increased close social contact with other adults; at adulthood, males showed an increased OTR density in the brain (Bales and Carter, 2003b; Kenkel et al., 2019). Of note, intranasal administration of OT to rhesus macaque neonates enhanced infants’ affiliative communicative gestures and reduced salivary cortisol; and higher levels of OT were correlated with more social interest. Infants with better imitative skills were most sensitive to the positive action of OT; it suggests that sensitivity to OT may underlie early social motivation (Simpson et al., 2014).

Among the genetic cases of ASD, SYS and PWS are relevant neurodevelopmental diseases that manifest feeding difficulties from birth, developmental delay/intellectual disability, and ASD (Figure 3). This is an interesting example showing a deficit in suckling activity at birth (see above), and alterations in cognition and social behavior in juvenile and adult stages (Meziane et al., 2015; Bertoni et al., 2021). *Magel2* KO mice show a decrease in mature OT release at birth, which is correlated with alterations of the onset of feeding behavior; the administration of OT just after birth restores a normal suckling (see above) (Schaller et al., 2010). In adult mutants, an alteration of OT-innervation and OTR expression was also reported (Meziane et al., 2015) and OT-treatment during the first week of life (one subcutaneous administration every day, starting just after birth) restores both, a normal behavior at adulthood and normalizes the OT-system. To further study the long-term effects of OT on adult behavior, our team focused on social memory that is impaired in male *Magel2-*KO mice. We showed that *Magel2* and *Otr* transcripts are co-expressed in the dentate gyrus and CA2/CA3 hippocampal regions involved in the circuitry underlying social memory. In *Magel2* mutants, we revealed: an increase of the GABAergic activity of CA3-pyramidal cells associated with an increase in the quantity of OTR-expressing cells, mainly somatostatin interneurons, in specific hippocampal regions and developmental timings. In *Magel2*-deficient pups, we also observed a delay in the GABAergic development sequence. Most importantly, we have demonstrated the therapeutical effects of subcutaneous administration of OT in the mutant neonates, restoring all hippocampal alterations and social memory at adulthood (Bertoni et al., 2021).

In conclusion, although the molecular mechanisms and neuronal circuits involved remain largely to be explored, evidence have clearly emerged to demonstrate that OT-treatment in neonates or during infancy, plays a determinant role in shaping the social behavior of the infants by controlling the activity-dependent development of brain structures, with consequences on the adult behavior.

## Conclusion and questions to address

**Figure 5:**
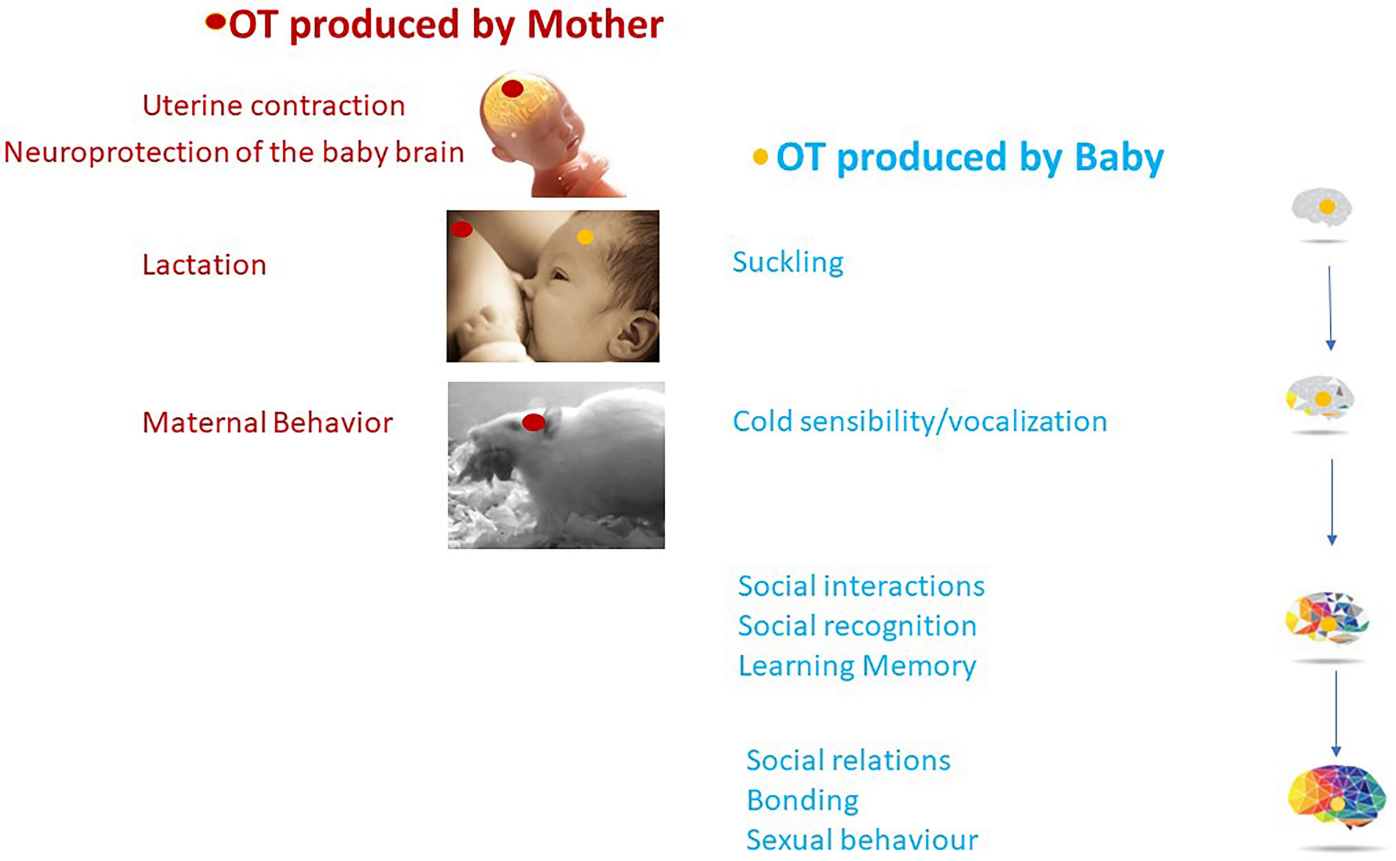
OT plays a concerted action between OT produced by the mother (in red) and OT produced by the baby (in blue) with long-lasting effects on social behavior and brain maturation.

The first sensorimotor and social behavior of a newborn mammal is to identify its mother, find the nipple, and suckle the milk. It is therefore not surprising that OT, by integrating sensory inputs in newborns, plays an important role in these processes. The OT-system is involved, from birth, in the first social interactions associated with feeding behavior on the mother’s side (lactation, suckling) and on the baby’s side (suckling) allowing the creation of a strong mother-infant bond. Thus, in addition to promoting the development of the sensory-motor system, OT has a concerted action in the mother and the baby, promoting the initiation of feeding behavior (Figure 5). How these two roles are related and dependent on OT circuits is illuminated by the pathologies in which both behaviors are affected.

Presumably, eating disorders in early childhood may lead to alterations in social behavior later in life. Early eating disorders could be an early marker of neurodevelopmental disorders such as ASD and could be an indication for early OT-treatment of these patients.

Considering the topic of this article, the main general questions to address in rodent models now are:

1. What sensory inputs affect OT-signaling in the first 2 weeks of life?
2. How does an identified sensory input affect the OT-system? In particular the expression of OTR (short and long term)?
3. What are the consequences, for an identified sensory input, of stimulation of the OT signal on sensory or motor responses?
4. What are the neural networks (functional connectivity) associated with a specific sensory stimulation involving the OT signal??
5. Does this OT signal play a role in the maturation of the OT-system?

These questions can be specifically applied to the role of OT in the initiation of sucking in rodents. To answer these questions, it would be necessary to develop efficient tools to study, around birth and in the first days of life, the connectivity of OT neurons and to manipulate OT neurons by optogenetics and chemogenetics.

In addition, studies to further analyze the alterations in early feeding and sucking behavior in ASD patients would be needed.

## Author contributions

FM designed and wrote the manuscript. VM and BC participated in the writing. All authors contributed to the article and approved the submitted version.

## Funding

This work was supported by the Foundation for Prader-Willi Research, Prader-Willi France, and Tonix Pharmaceuticals.

## Conflict of interest

The authors declare that the research was conducted in the absence of any commercial or financial relationships that could be construed as a potential conflict of interest.

## Publisher’s note

All claims expressed in this article are solely those of the authors and do not necessarily represent those of their affiliated organizations, or those of the publisher, the editors and the reviewers. Any product that may be evaluated in this article, or claim that may be made by its manufacturer, is not guaranteed or endorsed by the publisher.

## Footnotes

^1^ https://kimlab.io/brain-map/ot_wiring

^2^ https://kimlab.io/brain-map/OTR/
